# Dark ocean archaeal and bacterial chemoautotrophs drive vitamin B1 production in oxygen minimum zones

**DOI:** 10.1093/ismeco/ycaf077

**Published:** 2025-05-31

**Authors:** Kristin Bergauer, Christopher P Suffridge, Fabian Wittmers, Sebastian Sudek, Stephen J Giovannoni, Alexandra Z Worden

**Affiliations:** Ocean EcoSystems Biology Unit, GEOMAR Helmholtz Centre for Ocean Research Kiel, D-24148 Kiel, Germany; Department of Microbiology, Oregon State University, Corvallis, OR, 97331, United States; Ocean EcoSystems Biology Unit, GEOMAR Helmholtz Centre for Ocean Research Kiel, D-24148 Kiel, Germany; Marine Biological Laboratory, Woods Hole, MA, 02543, United States; Monterey Bay Aquarium Research Institute, Moss Landing, 95039, CA, United States; Department of Microbiology, Oregon State University, Corvallis, OR, 97331, United States; Ocean EcoSystems Biology Unit, GEOMAR Helmholtz Centre for Ocean Research Kiel, D-24148 Kiel, Germany; Marine Biological Laboratory, Woods Hole, MA, 02543, United States; Department of Geophysical Sciences, University of Chicago, Chicago, IL, 60637, United States

**Keywords:** ocean metabolomics, chemoautotrophs, foundational prototrophs, ammonia-oxidizing archaea, Nitrospina, Thioglobaceae, dark thiamine cycling, oxygen minimum zones

## Abstract

Vitamin B1 (thiamine) is essential for all cells, yet many marine microbes cannot synthesize B1 *de novo.* Dissolved thiamine and its related chemical congeners (TRCs) concentrations are not well characterized beyond the surface ocean, where they are typically low. Here, we observed unexpected enrichment of TRCs in regions of low dissolved oxygen levels (9.4 < O_2_ < 12.5 μmol kg^−1^) across vertical profiles in Monterey Bay and Pacific waters 145 km offshore (Station 67–70). TRC concentrations ranged from fM to pM, with 1.1 to 4.5 fold increases from near-surface waters to the mesopelagic Oxygen Minimum Zone (OMZ). Notably, at 67–70, dissolved B1 increased 3.5-fold within the mesopelagic OMZ. Paired metagenomic analysis suggests that chemoautotrophic ammonia-oxidizing Archaea (AOA) and Thioglobaceae, alongside nitrite-oxidizing *Nitrospina*, are important B1 producers in OMZs. Metagenome-assembled genomes indicate that *Nitrospina* may alternate between B1 biosynthesis and energy-preserving salvage pathways in synergy with co-occurring AOA. Re-analysis of metatranscriptomic reads from a previous study established Thioglobaceae can be dominant expressors of key *de novo* B1 biosynthesis genes in Monterey Bay. These findings suggest that deep ocean chemoautotrophs are B1 prototrophs in OMZs, analogous to photoautotrophs in the epipelagic ocean, and provide the foundation for B1 trafficking.

## Introduction

Marine microbial communities regulate energy conversion processes and affect climate and productivity by transforming organic and inorganic carbon molecules [1–3]. Recent attention has focused on the importance of B-vitamins in marine environments and their role in structuring communities, sparked by the realization that biochemically significant plankton are auxotrophic for one or more B-vitamins [4–7]. The necessity for either thiamine (B1 herein) biosynthesis or uptake is tied to its role as an essential coenzyme in primary carbohydrate and amino acid metabolism. B1 shortage can limit marine bacterial production and phytoplankton biomass [6, 8, 9].

**Figure 1:**
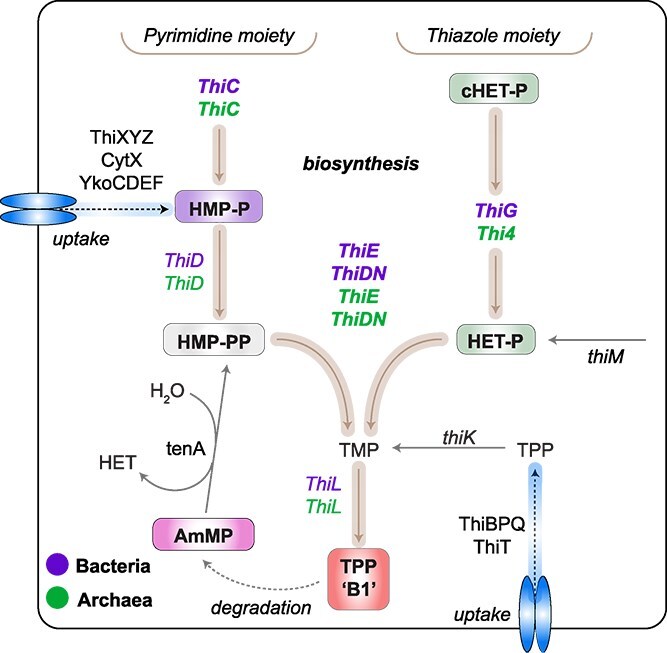
Thiamine biosynthesis in marine bacteria and archaea. Core biosynthesis (bold) and transport enzymes used by archaea and bacteria. In archaea, genes involved in thiamine biosynthesis are shown in green, whereas homologs in bacteria are indicated in purple. Abbreviations: *thiC* (hydroxymethyl pyrimidine synthase); *thiD* (hydroxymethyl pyrimidine (phosphate) kinase); *thiDN* (*thiD* fused with *thiN*; thiamine monophosphate synthase); *thiE* (thiamine phosphate synthase); *thiG*/*thi4*-type (thiazole synthase); thiL (thiamine phosphate kinase); *thiK* (thiamine kinase); *thiT* (thiamine; ABC transporter; ESF type); thiBPQ (thiamine; ABC transporter; type I ABC importer); *thiXYZ* (HMP; ABC transporter; type I ABC importer); *cytX* (HMP; putative secondary transporter; homologous to NCS1 family); *ykoCDEF* (HMP; ABC transporter; ESF type). Enzymes specific for salvage such as *thiM* (THZ kinase, EC 2.7.1.50), and *tenA* (aminopyrimidine aminohydrolase) are shown. Thiamine and pyrimidine and thiazole precursor compounds: HMP-P (4 aminohydroxymethyl-2-methylpyrimidine phosphate); HMP-PP (4-aminohydroxymethyl-2-methylpyrimidine diphosphate); TMP (thiamine monophosphate); TPP (thiamine pyrophosphate; herein B1); cHET-P (5-(2-hydroxyethyl)-4-methyl-1,3-thiazole-2-carboxylic acid phosphate); HET-P (4-methyl-5-(β-hydroxyethyl)thiazolium phosphate); and AmMP (4-amino-5-aminomethyl-2-methylpyrimidine).

Research on B1 trafficking at ocean depths below 200 meters—the dark ocean—remains sparse. The few measurements that exist indicate dissolved B1 concentrations are highly variable [10, 11]. In mesopelagic zones (200–1000 m), photosynthesis-based primary production is no longer feasible and the overall microbial metabolome changes substantially [12–16]. The *de novo* Thiamine Biosynthesis Pathway (TBP herein) includes biosynthesis of the pyrimidine (4-amino-5-hydroxymethyl-2-methylpyrimidine; HMP) and thiazole (4-methyl-5-2-hydroxyethyl thiazole; HET) moieties, followed by their condensation, which then creates the biologically active form of thiamine, after phosphorylation (Fig. 1). Analytical and metagenomic analyses support the conclusion that some algae [17] and abundant bacterial taxa depend on exogenous supplies of dissolved thiamine and/or its related chemical congeners (TRCs), including HMP and HET, because they lack the complete biosynthesis pathway [4, 17–20]. This type of vitamin dependency highlights the importance of trophic interactions between B1 auxotrophs and prototrophic microbes [21–23], like Cyanobacteria, Verrucomicrobiota and members of the Flavobacteria, which contribute to B1 production in epipelagic zones [24–26]. Uncertainties remain about the patterns by which B1 moves through food webs, such as mechanisms of transport (uptake and production), evolved interactions driven by cross-feeding, growth suppression by limitation, and how these patterns are affected by environmental conditions. A necessary next step is to identify net ``sources'' and ``sinks'' of dissolved TRCs [4, 18, 26] and possible effects of abiotic factors in the context of climate change. Dissolved TRCs have been quantified in surface waters, revealing generally trace availability (e.g. [4, 9, 11, 25, 27, 28]), but less is known about their distribution and cycling in the dark ocean, including oxygen minimum zones (OMZs) with low dissolved oxygen.

In the dark ocean, microbial processes are characterized by a diversification of energy sources [29] and a shift in the dominant taxa compared to the epipelagic layer [30]. The key primary producers in the dark ocean, chemoautotrophic microbes, utilize sulfide (and other inorganic sulfur compounds), ammonium, cyanate, and other electron donors (e.g. [31]) to accomplish inorganic carbon fixation. For example, the ammonia-oxidizing Archaea (AOA; i.e. Thaumarchaeota) are numerically abundant members of mesopelagic communities, that have a near monopoly on what might be the principal energy source for chemoautotrophy in the dark ocean [12, 32].

Naturally occurring OMZs with persistent hypoxia include regions below eastern boundary upwelling systems, such as Monterey Bay and silted basins like the Santa Barbara Basin. OMZs are dynamic environments shaped by episodic surface ocean productivity, creating hypoxic conditions (defined as dissolved oxygen being below 60 μmol kg^−1^) at around 300–350 meters depth as organic material sinks [33]. Members of the metabolically diverse Thioglobaceae (previously SUP05 clade), are widespread in mesopelagic zones and especially abundant in oxic-anoxic transition zones surrounding OMZs [34, 35]. Thioglobaceae support both heterotrophic and sulfur-based chemoautotrophic processes, utilizing sulfide and other forms of inorganic sulfur, and reduce oxygen, nitrate, nitrite, nitric oxide, or nitrous oxide to gain energy for inorganic carbon fixation via the Calvin Benson Bassham cycle [36, 37]. Conceptual models supporting the coupling of nitrogen- and sulfur-based energy metabolism with dark carbon fixation along redox gradients emphasize the tentative role of chemoautotrophic microorganisms as a source of new carbon in these regions [37, 38].

Here, we examine targeted metabolomics data and links to resident microbes based on genetic patterns of B1 biosynthesis, transport, and salvage across the vertical dimension. We measured dissolved TRC concentrations from epipelagic waters (2 m) to the benthic boundary layer (BBL; e.g., 3560 m with bottom depth 3562 m) in the eastern boundary upwelling system of the eastern North Pacific (ENP) off California, USA. Pronounced B1 accumulation was observed in the mesopelagic OMZ core and BBL. To shed light on the microbes and molecular mechanisms supporting “dark” B1 cycling we characterized community diversity and composition using 16S rRNA gene analyses and reconstructed B1 biosynthetic pathways in metagenome assembled genomes (MAGs) generated from Pacific Ocean sites sampled in this study. We leveraged publicly available metatranscriptomic data from vertical profiles transecting the same OMZ sampled prior to this campaign [39]. Metagenomic analyses identified chemoautotrophic Thioglobaceae, as well as dominant AOA and the often cooccurring nitrite-oxidizing *Nitrospina* (NOB), as potential principal producers of B1 in the dark ocean. In support of this finding, metatranscriptomic data highlighted the previously unrecognized role of sulfur-oxidizing Thioglobaceae in B1 biosynthesis. Collectively, our findings suggest that a trans-domain set of facultative chemoautotrophs are primary sources of B1 in OMZs. These results show parallels to surface ocean patterns, wherein select photoautotrophs appear to dominate vitamin B1 production. Collectively, our studies point to a potential evolutionary trend in which primary producers maintain thiamine prototrophy, allowing their autotrophic metabolism to occur when geochemical conditions are favorable, and reducing dependence on community interactions to maintain their supply of essential coenzymes.

## Materials and methods

### Oceanographic sampling

**Figure 2:**
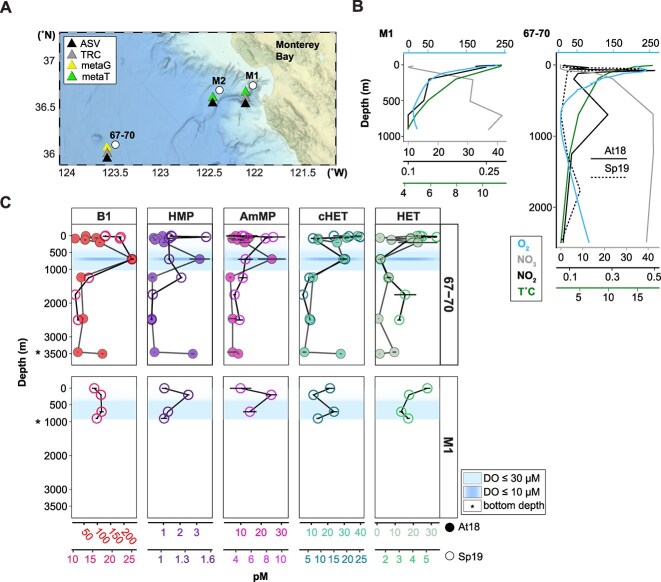
Concentrations of B1 and thiamine-related compounds (TRCs) along vertical oxygen gradients in the Pacific Ocean. (A) Location of the study sites (circles) in the ENP. The color of the triangles represents the type of sample analysis conducted, including ASV, vitamin 'TRC', metagenomic, and metatranscriptomic analyses. (B) Profiles of mean concentrations in μmol kg^−1^ of dissolved oxygen (DO, blue), μmol L^−1^ of nitrite (black; solid line autumn 2018 and dashed line spring 2019), μmol L^−1^ of nitrate (grey), and temperature (^o^C, green) at Stations M1 and 67–70. (C) Vertical profiles of dissolved TRC concentrations measured in the ENP at Stations 67–70 (top panel series) and M1 (bottom panel series) in autumn 2018 (closed circle) and spring 2019 (open circle). Separate x-axes are used to illustrate vertical trends in TRC concentrations in pM range; axis colors relate to the respective compounds. DO ≤30 μM is indicated in light blue shading (500–1250 m, at 67–70; 500–900 m, at M1) and the core of the OMZ, with DO ≤10 μM, is shaded in dark blue. Points and error bars represent means and standard deviations of technical replicates (n = 3). Asterisks denote the approximate position of the bottom depth.

Samples were collected using the *R/V Western Flyer* in August 2018 and April 2019. Seawater was obtained at Monterey Bay Time Series Stations M1 (36.446°N, 122.128°W) and M2 (36.688°N, 122.386°W, Fig. 2A), as well as 67–70 (36.712, 123.490), using Niskin bottles mounted on a CTD-rosette water sampler and the ROV Doc Ricketts for sampling the benthic boundary layer at 3510 and 3560 m in August 2018. CTD profiles and samples for measuring inorganic nutrients (Fig. 2B and Supplementary Data S1) were obtained alongside those for dissolved thiamine and precursor analyses as previously described [11]. Briefly, 1000 ml of seawater were filtered via a 0.2 μm Sterivex membrane (PES, Millipore) to remove cells and particles using an eight-channel peristaltic pump (flow-rate 30 ml min^−1^). The filtrate was collected in amber HDPE bottles (Nalgene) that had been acid washed and rinsed 3x with methanol. Samples were protected from light throughout the process. Samples for metagenomic analyses were collected from five depths (2 m, 60 m, 700 m, 1250 m, and 2500 m) during August 2018, and two depths (700 m and 2000 m) during April 2019 (Supplementary Data S2). Specifically, 40–75 L of seawater were sequentially pre-filtered over a 3 μm and 0.8 μm pore size Supor (Pall) membrane and collected onto 0.2 μm pore size membrane (PES, 142 mm diameter). Samples were flash frozen and stored at −80°C until extraction. For community composition analyses (16S rRNA gene amplicon sequencing), biomass from 500 ml of seawater was collected onto 0.2 μm pore size (47 mm diameter) Supor membranes, at 11 to 12 depth layers at Station 67–70, 6 depths at Station M2, and 8 depths at Station M1, and stored at −80°C.

### Quantification of Vitamin B1 and precursor compounds

Thiamine and its derivatives were extracted from seawater as previously described [11, 25]. Briefly, TRCs were extracted from the seawater matrix using solid-phase extraction with Bondesil C_18_ resin (Agilent). Salts were removed by washing with LCMS grade water and TRCs were eluted from the column using LCMS grade methanol. The resulting methanol-TRC eluate was evaporated to 250 μl using a blow-down nitrogen drier. LCMS analysis was conducted using an Applied Bioscience 4000 Q-Trap triple quadrupole mass spectrometer with an ESI interface coupled to a Shimatzu liquid chromatograph. Chromatography and mass spectrometry conditions as well as compound specific information including MRM parameters, column retention times, and limits of detection were described previously [25]. Quantification was conducted using ^13^C-labeled thiamine as an internal standard to compensate for matrix effects.

### DNA extraction for metagenomics

For high-molecular-weight DNA extraction from Station 67–70, cells collected onto 0.2 μm membranes were subjected to sucrose lysis followed by phenol:chloroform extraction as described previously [40]. Briefly, filter membranes were sectioned and incubated at 37°C for 60 min in lysis buffer (50 mM Tris(hydroxymethyl)aminomethane hydrochloride pH 8, 40 mM EDTA, 0.75 M sucrose, 1 mg ml ^−1^ lysozyme). After adding Proteinase K and sodium dodecyl sulfate to final concentrations of 0.5 mg ml ^−1^ and 1%, respectively, filters were incubated at 55°C for 120 min with gentle agitation. DNA was then extracted twice with phenol:chloroform:isoamyl alcohol (25:24:1) equilibrated to pH 8 and the resulting aqueous phase was purified on an AMICON-4100 kDa MWCO column following manufacturer’s protocol (Millipore). DNA fragment size was evaluated on a 0.7% agarose gel. DNA concentrations were measured with Quant-iT assay on a Qubit fluorometer (Invitrogen). Concentrations ranged from 0.6–4.5 ng μl^−1^ and 25 μl per sample was used as input for sequencing. Libraries were constructed using the Kapa HyperPrep kit (Roche) and sequencing was performed on a NovaSeq 6000 S2 (2 × 150 bp, Illumina).

### Sequence and data analysis

Data from seven metagenomic samples sequenced herein were processed as follows. Illumina raw reads were quality filtered using Trimmomatic (v.0.39; [41]), trimming after a base with a quality score below 3 or when the 25 bp moving average was <30. Reads were excluded from further analysis when <50 bp after trimming. On average, 44 341 428 ± 3 337 198 quality controlled paired-end reads were obtained from the Illumina NovaSeq platform. Samples were assembled separately using megahit (v.1.2.9; [42]) with the “—presets meta-sensitive” option and considering both paired and unpaired reads that passed quality control. The assembly was filtered using the ANVI’O script “anvi-script-reformat-fasta” (v.7; [43]) and only contigs longer than 5 Kb were retained for future analysis.

Bin taxonomy was estimated using GTDB-Tk (v.1.0.2; [44]) with default settings and GTDB version 214.1 as the reference database. Completeness and contamination estimates were computed using CheckM (v.1.1.3; [45]). Metagenomic bins were constructed using all versus all samples on MetaBAT2 (v2.12.1 [46]), based on tetranucleotide frequencies and differential coverage from sequence alignment maps generated with Bowtie2 (v2.4.1 [47]) using default settings and considering only paired-end reads for mapping to assemblies. Sequences that could not be assigned to a bin were retained for downstream analysis and annotation. We identified 677 MAGs before dereplication (Supplementary Data S3), comprising 132 medium- or higher quality MAGs (completeness ≥50%, and contamination ≤10%) representing 119 bacterial and 13 archaeal population genomes. These contained on average 12%, 21%, and 32% of each sample’s metagenomic reads from the epi-, meso-, and bathypelagic water layers, respectively. Genomes with an ANI of >95% to a seed genome, as calculated over the 120 bacterial or 122 archaeal marker genes used for phylogenetic inference, were clustered with that seed genome.

Prodigal (v2.6.3; [48]) was used for protein prediction, and functional annotations were obtained using prokka (v1.12; [49]). In addition, the blastKOALA and ghostKOALA tool servers [50] were used to obtain KO annotations for predicted proteins. Functional annotations were generated using hmmscan (v.3.3.2; [51], Table S1) with e-value ≤1^−10^ vs PFAM (v.35; [52]) and eggNOG-mapper (v.2.0.1; [53]) with the “-diamond” flag. For predicted proteins, hmmsearch was run with additional HMM profiles (B1-related biosynthesis, salvage and transporter proteins) obtained from TIGRFAM (v15.0) [54] and from [19] (Table S1, Supplementary Data S4). Protein annotations related to B1, nitrification and carbon fixation were further verified and filtered by a custom cutoff (e-value ≤1^−15^). Protein coding genes were analyzed on two levels to provide an understanding of B1 physiology (i) on contigs on assembly level and (ii) in all MAGs, before dereplication. For gene abundances, values were normalized to reads per kilobase million mapped reads (RPKM), reads that aligned with open reading frames in metagenomic assemblies.

### Metatranscriptome mapping

Quality-controlled reads from Stations M1 and M2 published in [39] were converted to paired read files using “reformat.sh” from BBMap (v.38.18; [55]) and recruited against each metagenome bin separately using Bowtie2 (v.2.4.1; [56]; removing unaligned reads and using the “—very-sensitive-local” alignment option). In addition, metatranscriptomic reads were mapped against selected JGI reference genomes (Supplementary Data S5) using the same approach. Bins and reference genomes were screened for functional domains relevant for B1 metabolism using hmmscan as described above. Sequence alignment maps were processed and compressed using samtools (v.1.6; [57]). Coverage per open reading frame was calculated using the bedtools “coverage” (v.2.30.0; [58]) subcommand with the “-counts” option. Coverage was normalized to rpkm (Supplementary Data S6).

### DNA extraction for amplicon sequencing and analysis

Nucleic acids were extracted using a DNeasy Plant kit (QIAGEN, USA) according to the manufacturer’s protocol, with modifications including a bead-beating step [59]. DNA was PCR amplified using the primer set 515F-Y (GTGYCAGCMGCCGCGGTAA) [60] and 926R (CCGYCAATTYMTTTRAGTTT) [61], targeting the V4-V5 hypervariable region of the 16S rRNA gene within Bacteria and Archaea. Briefly, 50 μl (total volume) PCR reactions were prepared with 5 μl of 10x buffer, 1 U of HiFi-Taq, 5 ng of DNA, and 200 nM primer (forward and reverse, respectively). PCR cycling parameters were 95°C for 2 min, 30 cycles of 95°C for 15 s, 55°C for 30 s, and 68°C for 1 min, with a final elongation set at 68°C for 7 min. Paired-end library sequencing (2x300bp) was performed using the Illumina MiSeq platform. Within QIIME2 [62], sequencing reads were processed by trimming primers using cutadapt [63] followed by quality filtering and denoising with DADA2 [64] (v1.10.0), generating amplicon sequence variants (ASVs). During the denoising step, forward and reverse sequences were trimmed to 250 and 220 bp, respectively. 218 044 ± 47 571 amplicons were sequenced per sample on average (Supplementary Data S7). After chimera check, amplicon taxonomy was determined using the assignTaxonomy command with the silva_nr_v123 database (Supplementary Data S8). 16S rRNA ASVs classified as chloroplasts were discarded.

### Statistical analyses

Data analysis was conducted in the R software environment [65]. Vegan package (version 2.6.4) was used for analyses of TRCs, RPKM and ASVs. Initial data visualization was conducted using the ggplot package (version 3.4.0) [66] followed by Adobe Illustrator (Adobe) for aesthetics. Differential abundance analyses were performed with DESeq2 [67]. The presence of indicator species (IndVal) analysis [68] was calculated using R software indicspecies (version 1.7.12) (Supplementary Data S13).

## Results

### Increased concentrations of dissolved TRCs suggest OMZs as B1 sources

To assess the concentration of metabolites produced via the TBP (Fig. 1), we quantified TRCs along vertical profiles at nearshore (M1) and offshore Pacific Ocean stations [66–69] using targeted metabolomics [11] (Fig. 2A). Dissolved TRC levels varied from femtomolar to picomolar at nearshore (M1: 15.43–17.35 pM B1, 4.61–8.63 pM AmMP, 1.04–1.33 pM HMP, 3.15–5.07 pM HET, 7.11–15.05 pM cHET) and offshore (67–70: 10.56–232.52 pM B1, 3.83–25.36 pM AmMP, 0.31–3.19 pM HMP, 0.34–22.07 pM HET, 3.05–38.33 pM cHET) sites when data across all depths and years were compared (Fig. 2C and Supplementary Data S1).

Trends in B1 distributions were consistent across the vertical dimension in the two seasons sampled at offshore Station 67–70. 2018–2019 B1 concentrations were on average lower in the upper 100 m of the water column (20.18–56.95 pM) and higher (19.56–136.09 pM) in the OMZ between 700–1250 m. A local maximum of 232.52 pM in the core of the OMZ (≤10 μmol O_2_ kg^−1^, Supplementary Data S1 and Fig. 2C) was observed in fall 2018. Below the OMZ, as DO increased, B1 levels declined (10.91–37.69 pM; Fig. 2C and Supplementary Data S1). Elevated B1 at 700 m coincided with higher concentrations of the B1 degradation product AmMP (25.36 pM), B1 precursors HMP (3.19 pM), and cHET in autumn 2018 (29.59 pM). In 2019 measured trends were similar in the vertical dimension but less pronounced.

### Evidence for B1 *de novo* biosynthesis in the Dark Ocean

To determine whether dark ocean microbes have the capacity for B1 and TRC biosynthesis that might explain our metabolomics results, we analyzed metagenomes from the sample set to identify thiamine-related metabolisms (Supplementary Data S9). On average, 4 162 226 ± 1 114 487 contigs were assembled per sample. To determine if OMZ taxa encode TBP genes more frequently than taxa from other depths we screened for *thiC*, *thiG*, and *thiE* of bacteria and *thiC*, *thi4*, and *thiDN* of archaea (Fig. 1, Supplementary Table S1), using representative HMM profiles [19]. Assigned HMMs scored 668 TBP protein sequences across all metagenomes (Supplementary Data S4), 46% of which encoded *thiE*, 29% *thiG*, 22% *thiC*, and 3% *thi4*-type synthases.

Relative TBP gene abundances based on the RPKM metric indicated an increase of *thiC* abundances below the epipelagic zone, suggesting prevalence of B1 prototrophy in the dark ocean (Supplementary Fig. S1A). Archaeal *thi4* gene abundances, which synthesizes the thiazole moiety of B1, were two to four times higher than bacterial homologues in meso- or bathypelagic zones (Supplementary Data S4). Similarly, archaeal *thiC* gene abundances were four times higher than bacterial counterparts in the bathypelagic zone (>1000 m), indicating archaeal contributions to prototrophy in the ocean’s interior (Supplementary Fig. S1B). Elevated AOA relative abundances (based on 16S rRNA amplicons) in mesopelagic zones observed here and by others in this region [70] supported this conclusion. Moreover, significant increases in *thiE* and *thiG* gene abundances from mesopelagic to bathypelagic zones (Kruskal-Wallis test, *P*-value <.01) demonstrate the bulk genetic potential for B1 synthesis. Differences in TBP gene stoichiometry, such as the higher *thiC* gene abundances, may reflect shifts in microbial community composition below the epipelagic zone. Similarly, multiple copies of *thiG* and *thiE* per genome have been reported before [19], further affecting gene stoichiometry.

### Putative B1 prototrophic and auxotrophic taxa in the eastern North Pacific

**Figure 3:**
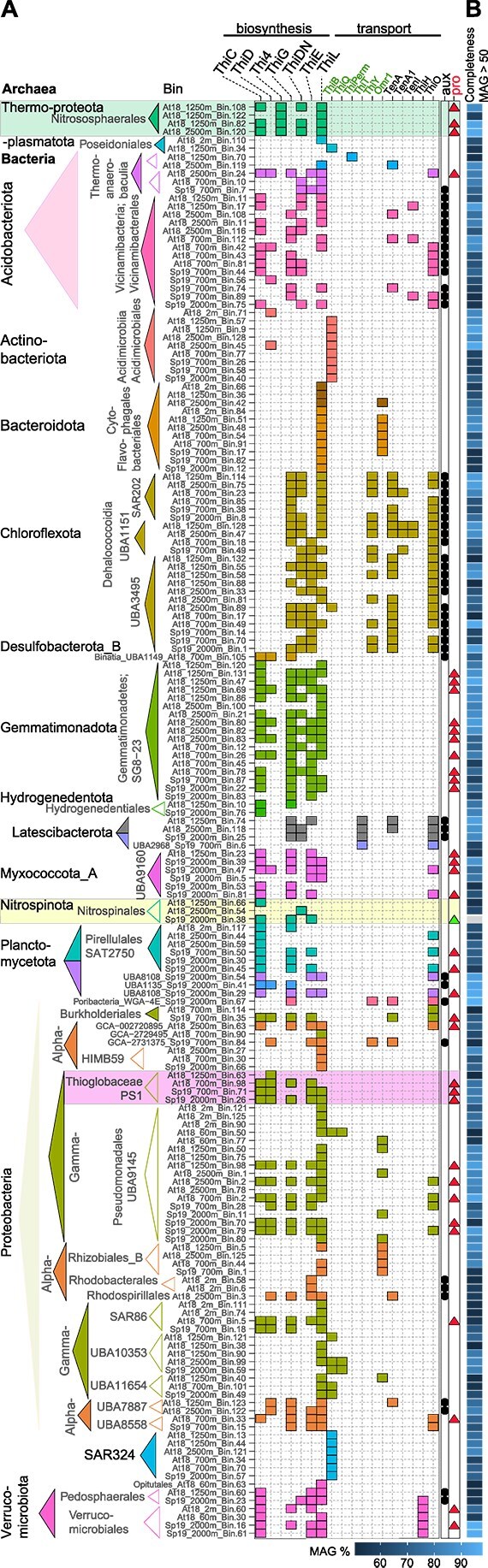
Thiamine metabolism potential of resident Pacific Ocean archaea and bacteria. B1-related genotypes of ENP MAGs. (A) The heatmap illustrates tentative B1 prototrophic and auxotrophic genotypes based on the presence (colored boxes) of key de novo synthesis and transport proteins in 174 medium-to high quality ENP MAGs (completeness ≥50%, contamination <5%) from offshore station 67–70. (B) Estimated completeness of ENP MAGs is indicated by the gradient of blue boxes on the right panel and MAG Bin ID’s with a completeness >90 are highlighted in bold letters. Taxonomy illustrates order-level assignment of MAGs via GTDB. Tentative prototrophic genotypes (pro) with synthesis genes *thiE*, *thiG* or *thi4*, and *thiC* are indicated by red triangles. Less conclusive data regarding putative pyrimidine (lack of *thiC*) and dual (lack of *thiC* and *thiG*) B1 auxotrophs (aux) are denoted with a black circle. *Nitrospina* Bin.38 (20% completeness indicated by a grey box) contained the full suite of TBP genes and is denoted with a green triangle.

We next inferred B1 physiology across MAGs assembled from Station 67–70 (Fig. 3A). Initial binning rendered 433 MAGs (dereplication ANI 95%), comprising 132 medium- or high-quality MAGs (completeness ≥50%, contamination ≤10%, Supplementary Data S3) representing 119 bacterial and 13 archaeal population genomes. Thiamine biosynthesis genes *thiCEG* or *thi4* were present in 11% of bacterial MAGs (n = 408) and 16% of archaeal MAGs (n = 25); affiliating with archaeal genera “*Nitrosopumilus maritimus”* and “*Nitrosopelagicus brevis”* (Fig. 3A, Fig. S2A and Supplementary Data S3) [71, 72]. The presence of both ammonia-monooxygenase (*amo*; Supplementary Data S10) and TBP genes in four archaeal MAGs suggests a synergistic potential for B1 prototrophy and chemoautotrophy in AOA populations.

B1 prototrophy signatures were identified across several bacterial phyla, including the Gemmatimonadota (n = 17), Myxococcota (n = 6), Nitrospinota (n = 2), Pseudomonadota (Thioglobaceae, PS1; n = 4), Planctomycetota (n = 10), Pseudomonadota (n = 17), and Verrucomicrobiota (n = 4). For some of these phyla, high quality MAGs (Fig. 3A-B) were reconstructed. Notably, two of the nine NOB MAGs assembled in this study (Supplementary Data S3) contained genes essential for B1 biosynthesis. The partial NOB MAG (Bin.38), did not meet the predefined completeness threshold (29% completeness, ≤1% contamination); however, it encoded all key biosynthetic genes (*thiCGE*) (Supplementary Fig. S2B) and was therefore included in the analysis. Additionally, to determine *Nitrospina’s* potential for B1 biosynthesis, we analyzed all NOB MAGs and conducted synteny analysis with reference genomes of closely related NOBs from aquatic habitats (Supplementary Data S11; Supplementary Fig. S2B). The presence of a complete B1 biosynthesis pathway in both ENP MAGs and NOB reference genomes (n = 4) suggests their ability to fully biosynthesize B1. Further, the co-occurrence of nitrite oxidoreductase (*nxr*) and signatures of B1 biosynthesis pathways within several NOB MAGs (Supplementary Data S10) indicates that these taxa play a dual role in both chemoautotrophic energy metabolism and thiamine production.

Thioglobaceae (PS1 clade) MAGs from the OMZ (700 m, Station 67–70) and deeper water layers possessed *thiCGE* genes, indicating that they can produce B1 (Fig. 2A). Several of these MAGs also encoded genes for adenylylsulfate reductase (*apr*), dissimilatory sulfite reductase (*dsr*), and ribulose-bisphosphate carboxylase (*rbc*; Supplementary Data S10), suggesting sulfur oxidation for energetic support of chemoautotrophic carbon fixation in these putative B1 prototrophs. These findings suggest that deep-ocean chemoautotrophs form the foundation of ocean-wide B1 economies.

Identifying B1 or precursor auxotrophy (i.e. encoding biosynthesis apart from one precursor moiety) is challenging due to MAG incompleteness (completeness ≥50%, Fig. 3B). With this caveat in mind, auxotrophic signatures were found in Acidobacteriota (class Vicinamibacteria), Chloroflexota (UBA1151, UBA3495, and SAR202), and lineages within Alphaproteobacteria (e.g. Rhodobacterales, Fig. 3A). Rhodobacterales (n = 2) lacked *thiC* and *thiG* but encoded *thiE*, enabling utilization of exogenous pyrimidine and thiazole precursors, consistent with prior results [19]. Dual precursor auxotrophs (i.e. taxa with just *thiE* and dependent on exogenous pyrimidine and thiazole precursors) were rarely assembled in metagenomes and were not further examined. Several UBA (Uncultivated Bacteria and Archaea) MAGs in the phylum Chloroflexota (n = 22), including UBA3495 (previously SAR202 group III; n = 12) and UBA1151 (formerly SAR202 group I; n = 4), lacked *thiC* (Fig. 3A), indicating pyrimidine auxotrophy. Subsequent analysis of 122 published MAGs and single amplified genomes of SAR202 [73] confirmed this dependency within SAR202. Interestingly, 65% of SAR202 genomes (Supplementary Data S12) possess *thiG* and *thiE*, pointing to a likely role in thiazole production in this metabolically versatile phylum. Some heterotrophic Chloroflexi representatives are known to rely on B1 breakdown products and transporters for cross-feeding (e.g. [74, 75]), further supporting their vitamin auxotrophic lifestyle.

B1 biosynthesis is frequently dependent on or augmented by networks of salvage enzymes that convert exogenous precursors into the active cofactor (Fig. 1). Thiamine monophosphate kinase (*thiL*) has a dual role functioning in both *de novo* synthesis and salvage and was identified in 60% of ENP MAGs. *ThiL* and a putative thiamine transporter (*omr1*) were encoded by Flavobacteriales (n = 8) and Cytophagales (n = 3) MAGs, indicating salvage, since key biosynthetic enzymes were not present. In line with its primary metabolic role in B1 biosynthesis, all AOA MAGs (completeness ≥50% and contamination <5%) possessed *thiL* (Fig. 3A). Enzymes specific for other B1 salvage reactions, such as *tenA* (conserved in Bacteria and Archaea), were present in 16% of MAGs, some of which were predicted to have various types of B1 auxotrophies (Fig. 3A). These include Chloroflexota and Latescibacterota lacking *thiC*, and Vicinamibacteria lacking *thiE*.

### Dominant OMZ community taxa are thiamine prototrophs

**Figure 4:**
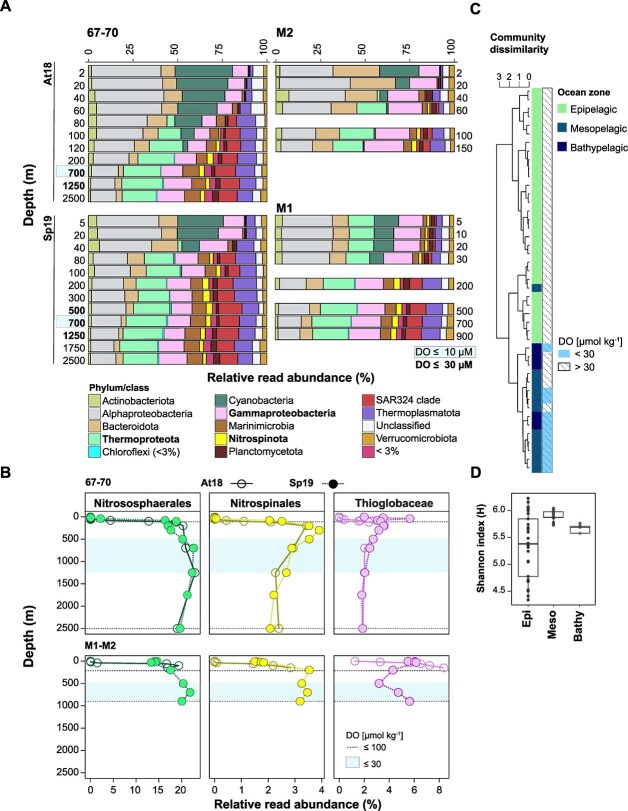
Microbial community composition patterns reveal depth-related shifts in contributions of newly identified B1 prototrophs. (A) Microbial community composition and relative amplicon abundance (% of total amplicons) of bacterial and archaeal phyla and major classes at Stations 67–70 (left panel), M1 and M2 (right panel) collected in autumn 2018 and spring 2019 cruises. Phyla accounting for less than 3% of amplicons were coined as “<3%” with the exception of the phylum Chloroflexi, accounting for <2%. The oxycline depths exhibiting DO ≤30 μM are indicated in bold, and the core of the OMZ (DO ≤10 μM) is shaded in blue. (B) Abundance profiles of newly identified B1 prototrophs. Values reflect percentages of the total amplicons observed during the autumn 2018 (open circles) and spring 2019 (filled circles) sampling campaigns at Stations 67–70, M2, and M1, respectively. Lines represent dynamics in vertical distribution in the seawater. DO levels are depicted following Fig. 2. (C) Cluster dendrogram showing the average linkage hierarchical clustering based on a Bray–Curtis dissimilarity matrix of amplicons sequenced in this study. Shading denotes amplicons from the epipelagic water layer (2–200 m) in green, from the mesopelagic (200–1000 m) light blue, and from the bathypelagic (1000–2500 m) dark blue. DO levels ≤30 μM in the respective water layers are indicated. (D) Diversity (Shannon index) of the microbial community of epi-, meso-, and bathypelagic water masses independent of sampling location or time of collection (autumn 2018 and spring 2019 combined).

To minimize biases from MAG assembly inefficiencies for microdiverse populations, we turned to community characterization using the 16S rRNA gene (V4-V5 region) (Supplementary Data S7 and S10). Epipelagic microbial communities were dominated by known groups in this region [39, 76], including auxotrophs and prototrophs (e.g. SAR11 and picocyanobacteria, respectively). Several SAR11 ecotypes were observed that are reportedly *thiC*-deficient and require exogenous HMP [4, 77], summing to 28% of total amplicon relative abundances. Diverse Gammaproteobacteria comprised 14% relative abundance, including SAR86, a B1 auxotroph ([26] Fig. 4A). Similarly abundant were tentative prototrophic and auxotrophic Flavobacteria groups (11%; NS2b, NS3a, NS4, NS5, and NS10) [19, 25, 78] and prototrophic Cyanobacteria formed 10% amplicon relative abundance [24]. Marine Group II (MGII) Euryarchaeota and Marine Group I (MGI) Thaumarchaeota reached 5% and 14% relative abundance, respectively at Station M1 (Fig. 4A-B). Extending into the mesopelagic OMZ (300–900 m), relative abundance of archaea increased, with Thaumarchaeota constituting ~20% and Euryarchaeota 9% of ASVs. Relative abundance of Marinimicrobia and SAR324 clade (average ~ 17%), SUP05 cluster Thioglobaceae (4%), and *Nitrospina*-like (3%) also increased (Fig. 3A-B). The relationships between TRCs, environmental parameters, and microbial community composition were examined using canonical analysis of principal coordinates (CAP; Fig. S3A). Observed richness estimates peaked at the top of the oxycline and decreased with increasing depth and DO (Supplementary Fig. S3B). Beta diversity analysis showed a typical depth-stratification (Fig. 4C), with alpha diversity highest in the upper mesopelagic layer (200–1000 m) as compared to the epi- or bathypelagic layers (Fig. 4D). A Wilcoxon rank sum test confirmed a significant shift in microbiome composition from epi- to mesopelagic water layers (*P*-value = .006).

Shifts in relative amplicon abundance of chemoautotrophic taxa that encode B1 biosynthesis capabilities were also observed (Fig. 4B). AOA, tentatively representing *Nitrosopumilus*-like ecotypes [79], were prevalent in epipelagic and mesopelagic waters at Station M1 (22% relative amplicon abundance). At 67–70 AOA increased with depth and accounted for up to 24% of amplicon abundance in the mesopelagic OMZ (300–900 m; Fig. 4A-B). Moreover, a prior analysis of thaumarchaeal ecotypes in Monterey Bay revealed that seasonality mostly influenced the distribution and abundance of ecotypes in the upper 50 m of the water column [70]. Here, NOB, typically widespread in some parts of the dark ocean (e.g. [80]), accounted for 1–4% of amplicon abundances across the water column, with highest relative abundances in the upper oxycline (200–300 m). NOB distribution patterns showed a distinct trend of co-occurrence with AOA (Fig. 4A-B), suggesting metabolic interdependencies. Chemoautotrophic Thioglobaceae accounted for highest relative abundances at Station M1 (≤8% of amplicons at 150 m depth). At M2, Thioglobaceae were enriched when DO was <30 μM (i.e. 900 m depth; Fig. 4B). Indicator Species Analysis (Indval) showed that signature species across the oxycline from 300–2500 m (in comparison to epipelagic microbes) belonged to the Planctomycetota, Verrucomicrobiota, Gemmatimmonadota, and Myxococcota (*P ≤* .001; Supplementary Data S13), all of which contain taxa that encode B1 biosynthesis pathways (Fig. 3A). AOA and NOB were not identified as OMZ indicator species (*P* = .009 and *P* = .005, respectively), likely because they were also present in M1 waters <200 m (Fig. 4A). The presence of multiple tentative B1 prototrophs, as determined by the gene content of MAGs herein, which contribute over 30% relative amplicon abundance in the OMZ microbial community, shows potential for active B1 production and cycling in oxygen-depleted, mesopelagic zones.

### High transcriptional activity of B1 biosynthesis genes in AOA and Thioglobaceae

We analyzed prior transcriptomic data (Supplementary Data S14; 39) to determine if B1 biosynthesis genes were actively expressed by the prototrophic cell types we identified in the Monterey Bay OMZ. The metatranscriptomes were collected in 2015 at depths comparable to our study at M1 and M2 (30–40, 80, 100, 150–200, 500 m; Supplementary Data S1 and S3), with low DO levels below 200 m (Fig. 2B); [39], and came from spring, like our Monterey Bay data. Therefore, the microbial community composition analyzed in [39] and here is likely highly comparable, as evidenced by similar depth-related patterns of AOA at M1 and M2 over a two-year period, with no significant seasonal influence observed [70].

**Figure 5:**
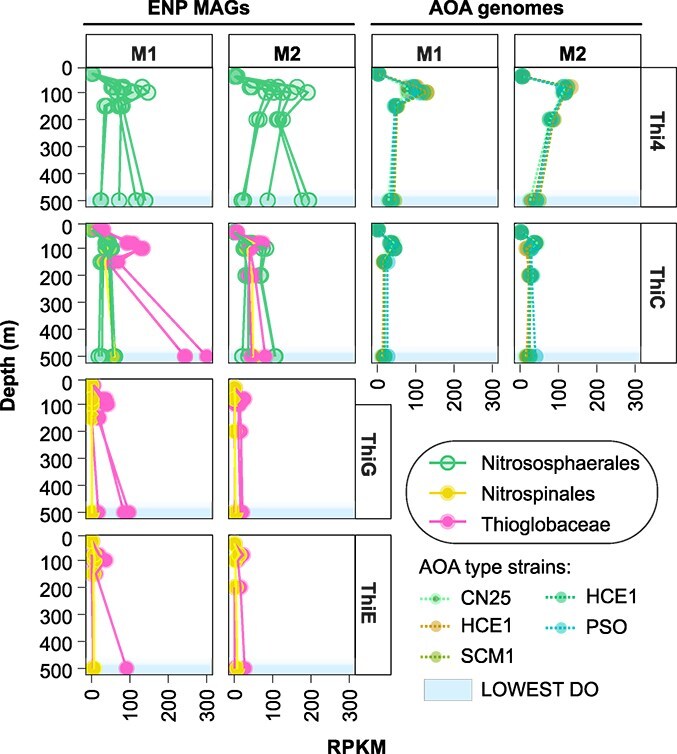
Relative expression and depth-dependent changes in transcript abundance of TBP enzymes along the oxycline. Vertical profiles of relative expression values in gene transcripts recruited against selected ENP MAGs and AOA reference genomes. ENP MAGs belong to the Nitrososphaeraceae, Nitrospinaceae, and Thioglobaceae. AOA reference genomes are type strains SCM1, CN25, HCE1, HCA1, and PSO (Supplementary Data S5).

Thus, we competitively mapped the metatranscriptomic reads against a larger selection of MAGs from our study (completeness >20%, contamination <5%; Supplementary Data S6). Class-level assignments demonstrated that MAGs belonging to Gammaproteobacteria and Nitrososphaeria recruited 40% and 22% of the thiamine-related transcripts, respectively, followed by Verrucomicrobiae (8%), Dehalococcoidia (6%), and Bacteroidia (5%), across the depth profile (Supplementary Data S6a). At M1, Thioglobaceae yielded highest RPKM values for thiamine-related reads (Fig. 5), recruiting the spring 2015 metatranscriptomic data against our spring 2019 MAGS. At M2, AOA dominated transcriptomic read recruitment at 100 m, 200 m, and 500 m, and accounted for 44%, 33% and 26% of thiamine-related expression, respectively (Supplementary Data S6b). At M1, Thioglobaceae had overall highest *thiC* mean expression values in the mesopelagic oxygen deficient water mass (500 m; Fig. 5), up to 4-fold higher than AOA. These results suggest that Thioglobaceae have a disproportional role in HMP and B1 production at this depth. In the mesopelagic zone, Thioglobaceae also accounted for highest *thiG* mean expression values, indicative of thiazole production, followed by MAGs of the orders UBA3495 (formerly SAR202 group III) and Pelagibacterales at 200 m depth (Supplementary Fig. S4A). While the published metatranscriptomes [39] were collected prior to the metagenomes presented in this study, the vertical expression trends for key biosynthesis genes aligned with our findings, confirming active expression of thiamine-related genes by identified taxa.

In addition to Monterey Bay partial MAGs (Supplementary Fig. S4A), we mapped M1 and M2 transcripts to genomes from five archaeal isolates sourced from public databases to interrelate activity to AOA type strains (Fig. 5 and Supplementary Fig. S4B). These isolates (SCM1, CN25, HCE1 HCA1, and PSO) have confirmed *de novo* B1 biosynthesis in culture [21]. Vertical expression profiles inferred from mapping reads to ENP MAGs showed that AOA transcription was lowest in the epipelagic layer and increased with depth, and had peak expression of key biosynthesis genes at 80 and 100 m in the oxygenated zone (Fig. 5). An increase of expression of *thi4* was also evident for some ENP MAGs in the OMZ at 500 m depth. TBP gene expression of AOA type strains mirrored that of archaeal ENP MAGs, however, with consistently lower expression values of *thi4* and *thiC* synthases (Fig. 5). AOA type strains accounted for equal shares of the transcript pool (~20%), with the PSO strain invoking marginally higher expression values at M1 (23%), indicating adaptations to low-oxygen environments [81].

At M1 and M2, NOB exhibited highest transcriptional expression at 80 m, accounting for 5% and 6% of total RPKM values. *Nitrospina* typically increase in abundance below 40 m in the Monterey Bay system [39], and account for >3% of the microbial community at 80 m (Fig. 4A). We did not correlate transcript data to relative abundance from 16S rRNA amplicons or environmental metadata of our study, given collection in different years. However, from a qualitative perspective, the vertical transcription profiles of Gammaproteobacteria and AOA (particularly, 'Nitrososphaerales' at the order level) in the 2015 dataset correspond with relative amplicon abundances of both phyla in this study.

## Discussion

Understanding the enzymatic and abiotic drivers that govern B1 bioavailability in the oceans is of great interest. Significant progress has been made in assessing oceanic B1 biosynthesis pathways (Fig. 1) and TRC distributions [11, 21, 28]. However, measurements often do not extend into meso-, bathypelagic, or benthic boundary layers [27, 25] where energy sources, communities, and microbial metabolism differ notably from the surface ocean. By assessing dissolved TRC concentrations in concert with the microbial community composition and MAGs (Fig. 2C, Fig. 3A, and Fig. 4A), we identified a previously undocumented pool of dissolved TRCs in the OMZ that appear to be produced by chemoautotrophic microbes prevalent in the mesopelagic ocean. Observed accumulation of B1 and related compounds suggests that ocean hypoxia, driven by climate change, could alter deep ocean B1 dynamics [82]. Our findings offer new perspectives on the dynamics, bioavailability and the production of this vital coenzyme across the vertical dimension in the ENP.

The enrichment of B1, HMP, AmMP, and cHET in the core of the OMZ indicates that chemical and metabolic differences in OMZs drive changes in thiamine cycling (Fig. 2C). In this study, TRC concentrations were higher in fall, suggesting that yet-to-be identified processes contribute to a net buildup (Fig. 2C). As expected, across all time points, strong vertical gradients existed in physicochemical variables (Supplementary Fig. S5A-E). Both biotic and abiotic factors influence TRC availability; however, most proposed mechanisms, described below, are derived from studies conducted in the epipelagic and oxygenated zones [5, 69, 83, 84]. Factors affecting B1 metabolism include key microbial B1 producers, such as cyanobacteria and diatoms, which are fueled by sunlight [17, 20, 85], as well as B1 degradation due to UV radiation exposure [86]. In the mesopelagic and bathypelagic zones, additional environmental parameters [87] and oxygen availability may also influence microbial thiamine metabolism and reservoir dynamics. Favorable redox conditions [83, 84, 88] may enhance the chemical stability of dissolved TRCs. Given that the primary source of these compounds is biological activity, we hypothesize that elevated concentrations within OMZs could result from local biosynthesis by dominant microbial communities adapted to these low-oxygen environments. Studies have shown that OMZs harbor unique microbial assemblages capable of thriving under hypoxic conditions [35–38], potentially contributing to localized thiamine production.

**Figure 6:**
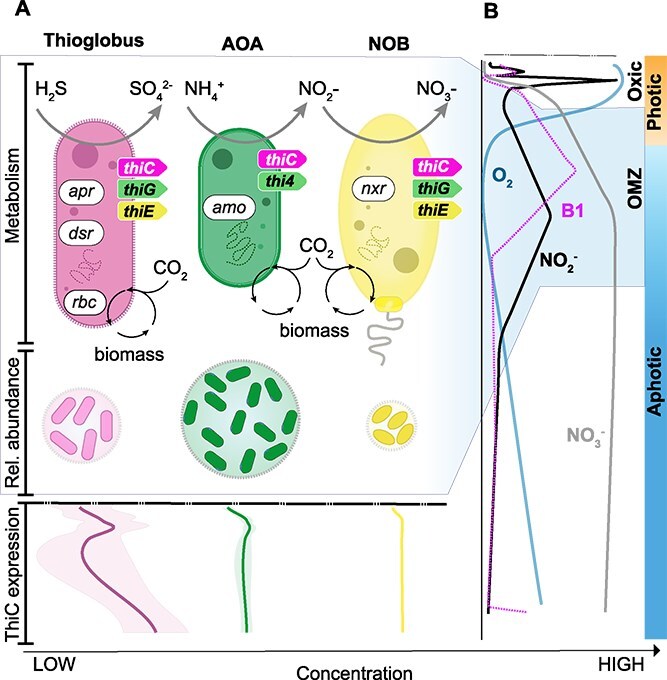
Conceptual sketch of the autotrophic and prototrophic potential across the OMZ. (A) Top: Illustration of metabolic traits of AOA, NOB, and Thioglobaceae MAGs on the basis of key enzymes indicative of denitrification and nitrification, dissolved inorganic carbon fixation, and B1 de novo synthesis. Archaeal *amo*, ammonia-monooxygenase (K10944); bacterial *nxr*, nitrite oxidoreductase (K00371; *narH*); *apr*, adenylylsulfate reductase (K00394, K00395); *dsr*, dissimilatory sulfite reductase (K11180); *rbc*, ribulose-bisphosphate carboxylase (K01601). Bottom: Relative abundance (size of circles) of tentative B1 prototrophs aligned with transcription of *thiC* expression, indicative of de novo synthesis. (B) Water column structure (curves for nitrogen species, oxygen and B1) along oxic/anoxic and photic/aphotic zones in connection to the distribution of dissolved B1 concentrations. See also Dataset S10.

Metagenomic analyses suggest that prevalent chemoautotrophic taxa within the OMZ are B1 prototrophs and are presumably responsible for the majority of dissolved TRC production in this environment (Fig. 6A-B). To provide culture-independent genetic evidence supporting the capacity of prolific and widespread AOA and other chemoautotrophs to synthesize B1 *de novo* (Supplementary Fig. S2A-B), we investigated biosynthetic gene cluster synteny - providing the genomic context of thiamine metabolism genes in our novel B1 prototrophs. Possession of a *de novo* B1 biosynthetic route allows independence from exogenous TRC sources, though it incurs an energetic cost. Chemoautotrophs like ammonia-oxidizing Archaea, nitrite-oxidizing *Nitrospina*, and sulfur-oxidizing members of the Thioglobaceae are crucial for nitrogen and sulfur cycling, as well as dark carbon fixation. B1 is essential in the Calvin Benson Bassham cycle for catalyzing the rearrangement of a 2-carbon chain into a pentose through its interaction with transketolase, as performed by organisms like the Thioglobaceae. The genus *Nitrospina* uses B1 in the reductive TCA cycle to facilitate the decarboxylation of two distinct 2-oxoacids into their corresponding CoA derivatives through the pyruvate oxidoreductase enzyme. Interestingly, B1 does not serve as a cofactor in the 3-hydroxypropionate/4-hydroxybutyrate cycle, which is the carbon fixation route used by AOA.

AOA are fundamental yet overlooked drivers of B1 trafficking in the mesopelagic. This assertion is based on recent biochemical evidence of cellular B1 production by AOA [81], and their high relative abundance at stations occupied in this study (Fig. 4A). The synthetic potential to produce B1 is well-established in the domain of Archaea (reviewed in [89]), but the significance and ecological relevance of AOA (i.e. Thaumarchaeota) in marine B1 cycling have received little attention. We demonstrate that the archaeal key biosynthetic genes *thiC* and *thi4* were highly expressed below the epipelagic zone (Fig. 5) in metatranscriptomic data [39]. This indicates that AOA likely play a substantial role in the observed enrichment of dissolved B1 in the OMZ, with repercussions upon auxotroph survival and B1 trophic transfer. Moreover, AOA have been linked to vitamin B12 (cobalamin) production in marine environments [90], and metabolites such as B2 (riboflavin), B6 (pyridoxin), B5 (pantothenic acid), and B12 have been confirmed in archaeal cell cultures [21, 81, 91]. Together these findings suggest a significant role of AOA in B-vitamin cycling, and rates of production, use, and exchange deserve particular attention given the ubiquity of AOA [13, 32, 92, 93].

NOB MAGs encoded *thiCEG*-key genes of the TBP as well as “salvage-related” gene clusters (*tenA-thiD*), suggesting flexibility in B1 metabolism and the potential for B1-mediated microbial interactions within this group (Supplementary Fig. S2B). The clustering of *tenA* with *thiD* in *Nitrospina* is an intriguing pairing of what is considered a degradative (*tenA*) and a biosynthesis function (thiD). We speculate that NOB use *tenA* to recycle AmMP into HMP, which can then be reintroduced into the B1 cycle (Fig. 1). This suggests flexibility between B1 production and energy-preserving pyrimidine/AmMP scavenging, potentially in metabolic exchanges with co-occurring AOA, which likely enhances competitiveness in fluctuating nutrient environments. Marine *Nitrospina* are reportedly cobalamin (B12) auxotrophs, relying on external sources, possibly supplied by other hetero- or autotrophic organisms including AOA [94], highlighting the interactive nature of this group. Despite their typically low abundances [95], single-cell analyses demonstrated that *Nitrospina* are active in inorganic carbon fixation in the mesopelagic [15]. Interestingly, their large cell size or suggested high mortality may explain low recovery in metagenome assemblies and amplicon datasets compared to AOA [15, 95]. Our concept of trophic interaction with AOA extends beyond reciprocal feeding on nitrogen-rich substrates [15, 91], considering key growth factors beyond nitrogen. In summary, our results indicate that *de novo* synthesis of B1 is highly regulated and that salvage routes that utilize degradation products [5, 6, 18, 75] open possibilities for niche partitioning and cooperation across taxa.

Members of the Thioglobaceae contribute to B1 production along the oxycline, dominating *thiCGE* gene expression at M1 (Fig. 5). This group is abundant in low-oxygen waters in upwelling regions [96, 97], playing critical roles in dark carbon fixation, denitrification, and sulfur oxidation [98]. The observed co-occurrence of RuBisCO (*rbc*L type II), *apr*, and *dsr* genes in these tentative B1 prototrophic MAGs implies that chemoautotrophic carbon fixation may be energetically supported by dissimilatory sulfur oxidation (Dataset S10). Whether these two processes are intimately linked warrants further investigation.

Overall, our findings indicate an unbalanced B1-based economy in the mesopelagic, specifically within OMZs (Fig. 6). The observed enrichment of dissolved TRCs in OMZs and omic-based analyses highlight chemoautotrophs as key B1 prototrophs. We propose that low oxygen levels may have led to adaptations in metabolic strategies or changes in ecological interaction rates or redox chemistry. This is evidenced by NOB’s apparent ability to alternate between biosynthesis and uptake, causing a decoupling of sources (B1-related compounds, new production) and sinks (salvage, degradation, and export). Our observations suggest that trophic cascades based on autotrophy include vitamins. While primary producer roles in controlling nitrogen availability in the dark ocean are established, extending this principle to vitamin B1 redefines our perception of B1 traffic as a recycling economy in which heterotrophic prototrophs may contribute, but autotrophs appear to be the dominant players.

## Supplementary Material

ISMEcomm-SI_proofed

ISMEcommun_SI_datasets_proof_30May2025

## Data Availability

The complete TRC dataset is presented in Supplementary Data S1. Metagenomic sequences, assembled MAGs, and amplicon data presented in this study are deposited in figshare (10.6084/m9.figshare.29133767) or at NCBI under BioProject PRJNA1023546.
