# Supplementary material for: Dark ocean archaeal and bacterial chemoautotrophs drive vitamin B1 production in oxygen minimum zones
Source: ISME Commun. 2025 May 31;5(1):ycaf077. doi: 10.1093/ismeco/ycaf077 (PMC12236431; doi:10.1093/ismeco/ycaf077)
Supplement: ISMEcomm-SI_proofed [file ismecomm-si_proofed.pdf]

Supporting Information for

Dark ocean archaeal and bacterial chemoautotrophs drive vitamin B1 production in oxygen minimum zones

Kristin Bergauer[1], Christopher P. Suffridge[2], Fabian Wittmers[1,3], Sebastian Sudek[4], Stephen J. Giovannoni[2] & Alexandra Z. Worden[1,3,5*]

[1]Ocean Ecosystems Biology Unit, GEOMAR Helmholtz Centre for Ocean Research, Kiel, 24148 Germany

[2]Department of Microbiology, Oregon State University, Corvallis OR, 97331 United States

[3]Marine Biological Laboratory, Woods Hole MA, 02543 United States

[4]Monterey Bay Aquarium Research Institute, Moss Landing CA, 95039 United States

[5]Department of Geophysical Sciences, University of Chicago, Chicago IL, 60637 United States

This file includes:

Supporting text

Figures S1 to S5

Table S1

Legends for Datasets S1 to S14

Supporting References

Supporting Information Text

Supplementary Methods

Synteny Plot

Genome synteny was visualized using ggplot2 (v.3.4.0; (1)) and the genomes expansion (v0.9.5.90; github: https://github.com/thackl/gggenomes) in R. Using prodigal and hmmscan, open reading frame coordinates for both reference genomes and metagenome bins were predicted, and selected thiamine metabolism-related genes (*thi4, thiC, thiG, thiS* and *TMP-TENI*) annotated as described above. Homology between genomes was computed using blastP (v.2.2.31; (2)). Homology is only indicated to adjacent genomes, requiring a blastP hit with an e-value $\leq 10^{-10}$ and requiring a minimum pairwise sequence identity of 40%. Contigs were trimmed 4 kb upstream and downstream of a thiamine-metabolism-related gene or at the end of a contig. Loci encoding at least one thiamine-metabolism-related gene were merged when the distance between the two loci was below 10 kb. ORFs encoding an *AirS* domain were only included in the synteny when encoded within 10 kb of another thiamine-metabolism-related gene. Genomes were ordered according to the tip order in phylogenomic trees for the bacterial and archaeal genomes separately. Trees were built using gtdb-Tk (v.1.4.1; (3)). Initially, single-copy marker genes were identified using ‘gtdbtk identify’ and aligned and concatenated using ‘gtdbtk align’, both with default settings. Then, phylogenomic trees were constructed using ‘gtdbtk infer’, using the constructed concatenated sequence alignments for the archaeal and bacterial genomes respectively.

Supplementary Results

Sample location and abiotic parameter

Stations M1, M2, and 67-70 extended out from the Monterey Bay canyon (21-145 km offshore; Fig. 2a), where Dissolved Oxygen (DO) concentrations decreased rapidly to ≤20 µmol kg^{-1} at M1 and M2 (bottom depth <1100 m) by 450-880m, and at 67-70 (bottom depth <3565 m) by 550-1050 m, below which DO concentrations increased (Fig. 2b; Supplementary Data S1). Accumulations of nitrite at stations M1, M2, and 67-70 occurred at the deep chlorophyll maximum (DCM; ~0.5 µmol L^{-1}) ranging between 40–80 m, and at depth (700 and 1750 m, respectively; Supplementary Data S1). Pronounced secondary nitrite maxima (SNM; >1 µM) resulting from anaerobic nitrate reduction to nitrite under low DO were not recorded. In the present data, surface waters in late September at offshore 67-70 were warm (18-11˚C) relative to the upwelling period (13-9˚C to ∼100 m; Supplementary Fig. S5A-E), marking the transitional period between upwelling and winter seasons (4, 5). Surface nitrate levels in spring and fall remained low (<1 µM) at offshore station 67-70 relative to coastal station M1 (>10 µM), occupied during spring (predicted upwelling season).

Pathways of thiamine synthesis vary among dominant OMZ lineages

To more robustly explore potential biosynthesis capabilities, we examined the syntenic status and conservation of B1 *de novo* synthesis and resolved the thiamine pathway architecture across selected ENP MAGs (n=15) and relevant

AOA (7 marine isolates) and bacterial (4 *Nitrospina* and 3 Thioglobaceae) reference genomes (Supplementary Fig. S2A and B, and Supplementary Data S13). The B1 biosynthetic gene complex of AOA was complete but differed markedly from NOB and Thioglobaceae genomes (Supplementary Fig. S2B). All analyzed AOA (including ENP MAGs) possess homologues to the molecular hallmark enzymes *thiC* and *thiDN* (analogous to *thiE* in Bacteria) and have replaced the canonical cHET biosynthetic branch with a *thi4* thiazole synthase, thereby encoding a complete *de novo* TBP. The three core genes involved in B1 biosynthesis were scattered on the genome and formed a primary region containing *thiC*-*thiDN* homologs and a second single gene locus encoding *thi4*. The *thi4*-type thiazole synthase, as well as the accessory sulfur carrier protein *thiS* and the thiamine phosphate kinase *thiL* genes, were several genes to >100 kb apart. This gene arrangement was conserved across closely related marine AOA genomes as well as the archaeal MAGs analyzed here. All AOA genomes and MAGs (except one, likely due to assembly issues) encode *tenA*, which could alleviate coenzymic requirements. *ThiC* amongst AOA shared 84–99% amino acid identity (AAI), whereas archaeal and bacterial *thiC* had much lower AAI (39–44%). *Nitrososphaera viennensis* EN76, a soil AOA, additionally encoded a putative hydroxymethylpyrimidine transporter *cytX,* just upstream of *thiC* (Supplementary Data S14), suggesting that some AOA can scavenge HMP.

Interestingly, *Nitrospina* MAGs and reference genomes encoded a canonical B1 biosynthetic complex where key genes *thiCEG* are syntenic (Supplementary Fig. S2B). Surveyed NOB genomes encode the accessory *thiS*

protein located directly upstream of the biosynthetic complex, serving as a sulfur donor for the *thiG*-mediated formation of the thiazole ring. Furthermore, we identified a ‘salvage-related’ or ‘regeneration’ complex with spatially co-located bifunctional *thiD* kinase (EC:2.7.1.49 2.7.4.7) and *tenA* (thiaminase II; EC:3.5.99.2), indicating potential co-transcription. *TenA* has been shown to play a dual role in B1 metabolism (e.g., in *Staphylococcus aureus*) (8), catalyzing the hydrolysis of thiamine to give HMP and HET (that is degradation), as well as the conversion of 4-amino-5-aminomethyl-2-methylpyrimidine (AmMP) to form HMP (that is regeneration) (9), which can be further phosphorylated by *thiD*. The *tenA*-*thiD* homologues are coded away from the *thiCEG* cluster but were syntenic in strains NAT221, NAT278, and *N. gracilis* 3/211. ENP MAG ‘Bin.66’ *thiC* gene closely affiliated (AAI 96%) with the environmentally relevant candidate genus “*Nitromaritima*” (Nitrospinae Clade 1). A recent study has suggested that members of this clade are the main drivers of nitrite oxidation in the global oceans and might play a key role in dark carbon fixation (10). A high-affinity ABC transporter implicated in thiamine import (*thiBPQ*) was encoded in ENP *Nitrospina* MAG ‘Bin.52’.

In Ca. *Thioglobus autotrophicus*, Ca. *Pseudothioglobus singularis* and affiliated MAGs, we found *thiCGE* genes scattered throughout the genome and encoded on single loci (Supplementary Fig. S2B). Other enzymes specific to salvage, such as *ThiM* (kinase; EC 2.7.1.50), or degradation of B1 (e.g., thiaminase I; EC 2.5.1.2) were not detected in the above-mentioned microbial species.

Supplementary Figures

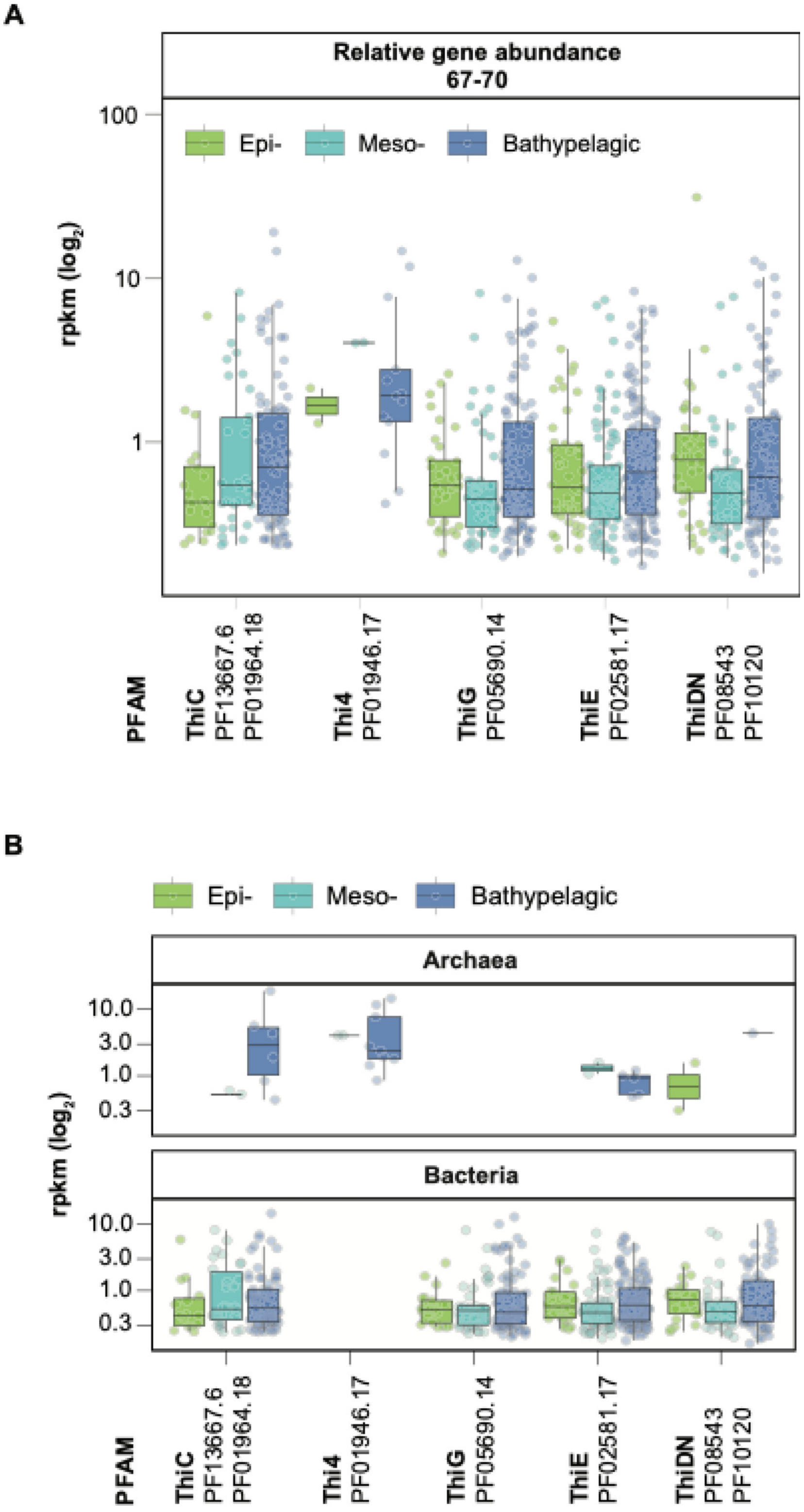

Fig. S1. Vertical trends in relative gene abundances of essential B1 biosynthesis genes. A) Mean relative gene abundances (station 67-70) in rpkm (log scale) for **B)** bacterial and archaeal *thiC* (PF13667.6), *thi4* (PF01946.17), *thiG* (PF05690.14), *thiE* (PF02581.17), and *thiDN* (PF08543; PF10120) are shown for epi-, meso-, and bathypelagic water layers. Vertical distribution patterns are highlighted by a unifying color scheme for metagenomes: green - epipelagic ~2m, 60m; light blue - mesopelagic ~700 m; dark blue - bathypelagic ~1250 m, 2000 m, 2500 m.

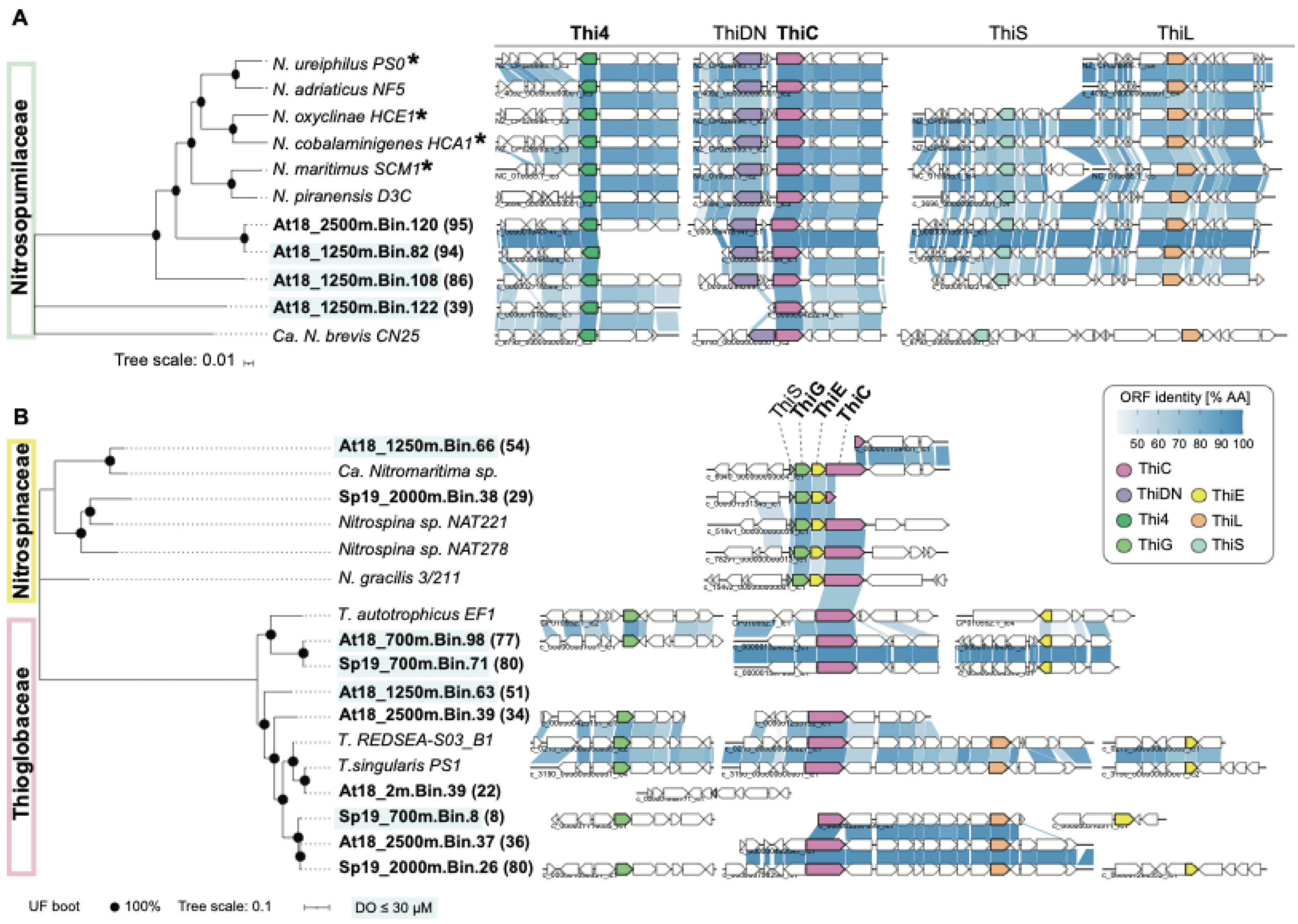

A
Thi4
ThiDN
ThiC
ThiS
ThiL
Nitrosopumilaceae
N. ureiphilus PS0*
N. adriaticus NF5
N. oxyclinae HCE1*
N. cobalaminigenes HCA1*
N. maritimus SCM1*
N. piranensis D3C
At18_2500m.Bin.120 (95)
At18_1250m.Bin.82 (94)
At18_1250m.Bin.108 (86)
At18_1250m.Bin.122 (39)
Ca. N. brevis CN25
Tree scale: 0.01
B
ThiS
ThiG
ThiE
ThiC
ORF identity [% AA]
50 60 70 80 90 100
ThiC
ThiDN
ThiE
Thi4
ThiL
ThiG
ThiS
Nitrospinaceae
At18_1250m.Bin.66 (54)
Ca. Nitromaritima sp.
Sp19_2000m.Bin.38 (29)
Nitrospina sp. NAT221
Nitrospina sp. NAT278
N. gracilis 3/211
Thioglobaceae
T. autotrophicus EF1
At18_700m.Bin.98 (77)
Sp19_700m.Bin.71 (80)
At18_1250m.Bin.63 (51)
At18_2500m.Bin.39 (34)
T. REDSEA-S03_B1
T.singularis PS1
At18_2m.Bin.39 (22)
Sp19_700m.Bin.8 (8)
At18_2500m.Bin.37 (36)
Sp19_2000m.Bin.26 (80)
UF boot
100%
Tree scale: 0.1
DO ≤ 30 μM

127

Fig. S2. B1 biosynthesis architecture and associated genes in ENP MAGs and reference genomes of OMZ dominants. Gene order and synteny of pyrimidine (*thiC*), thiazole (*thiG, thi4*-type), thiamine phosphate (*thiE*) synthase homologues and auxiliary thiamine metabolism genes identified in ENP MAGs (bold) and reference genomes (JGI Scaffold). Gene organization of **A)** archaeal contigs of selected type strains and archaeal ENP MAGs and **B)** bacterial contigs of isolates and bacterial ENP MAGs (Supplementary Data S4). Gene map similarity lines represent reciprocal BlastP hits with an e-value $\leq 10^{-5}$ and percentage ORF identity as shown in the upper-right legend. Ultrafast (UF; [109]) bootstrap support is indicated by circles. Asterisks indicate archaeal strains reportedly producing B1 in culture. Blue shading indicates OMZ MAGs. Numbers in parentheses denote completeness in %.

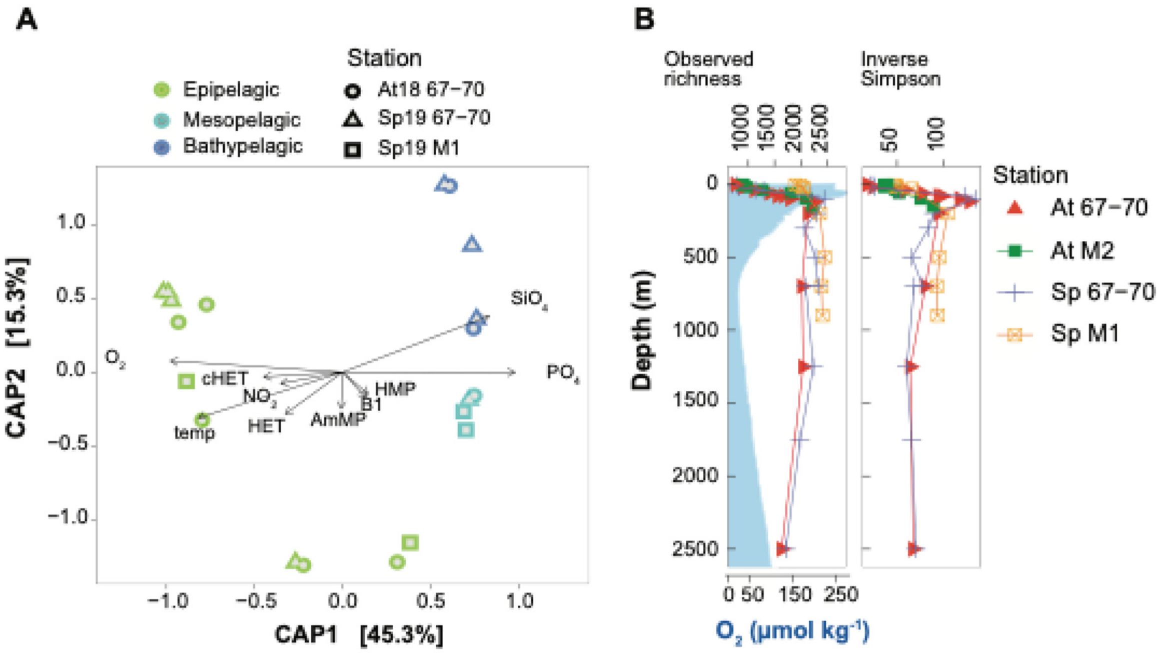

Fig. S3. Canonical Analysis of Principal Coordinates (CAP) of 16S rRNA ASV profiles constrained by physical and chemical environmental parameters, including TRC measurements and diversity indices grouped by water layers. A) Canonical Analysis of Principal Coordinates (CAP) of 16S rRNA ASV profiles constrained by physical and chemical environmental parameters, and B1 and precursor concentrations measured in this study. Ordination was constructed with the Bray–Curtis distances among samples. Choice of shape delineates the origin of the ASV library (station) and colors correspond to designated water masses (epipelagic – yellow, mesopelagic – red, bathypelagic – brown). The axis represents the first and second constrained component and the percentages correspond to the fraction of the variance explained under the linear combination of the selected environmental parameters. Arrows show the variance direction of each environmental parameter. Note, at station M2, samples were collected exclusively from the epipelagic layer and thus excluded from CAP

analysis. **B)** Depth profiles of Observed richness and evenness (Inverse Simpson) at the four sampling sites. Mean DO concentrations (μmol kg^{-1}) are illustrated in the left panel and correspond to the lower x-axis. Data are from normalized libraries from station 67-70 (autumn depicted in red, spring in blue), M1, and M2.

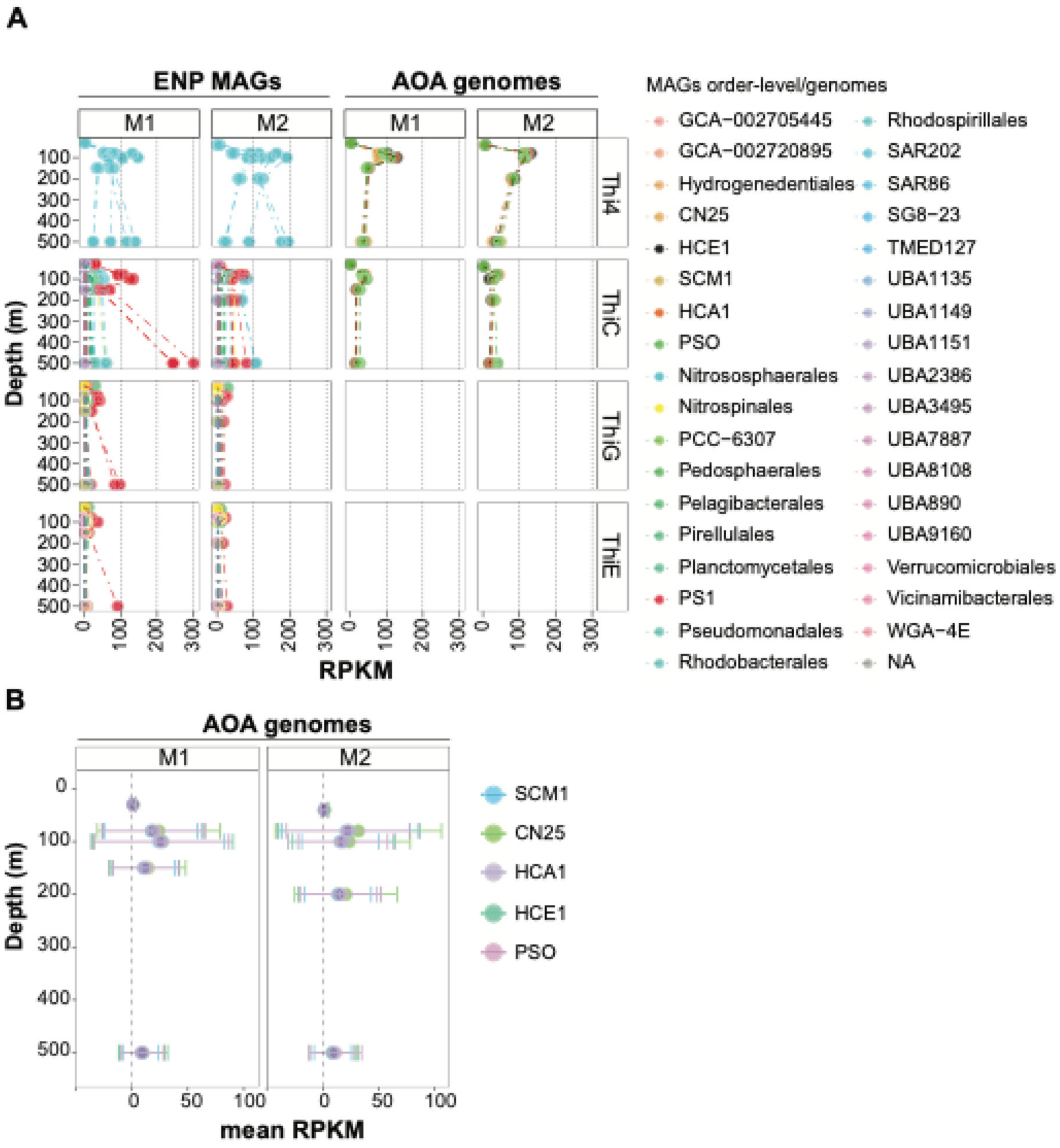

Fig. S4. Comparison of expression levels of *de novo* thiamine biosynthesis genes along across the water column. A) Vertical profiles of relative expression values (rpkm) of *thiC*, *thi4*-type, *thiG*, and *thiE* gene transcripts recruited against all ENP MAGs, resolved at the order level, and AOA reference genomes. **B)** Mean expression values (rpkm) of experimentally verified B1 – producing AOA strains, aligned against metatranscriptomic libraries (6).

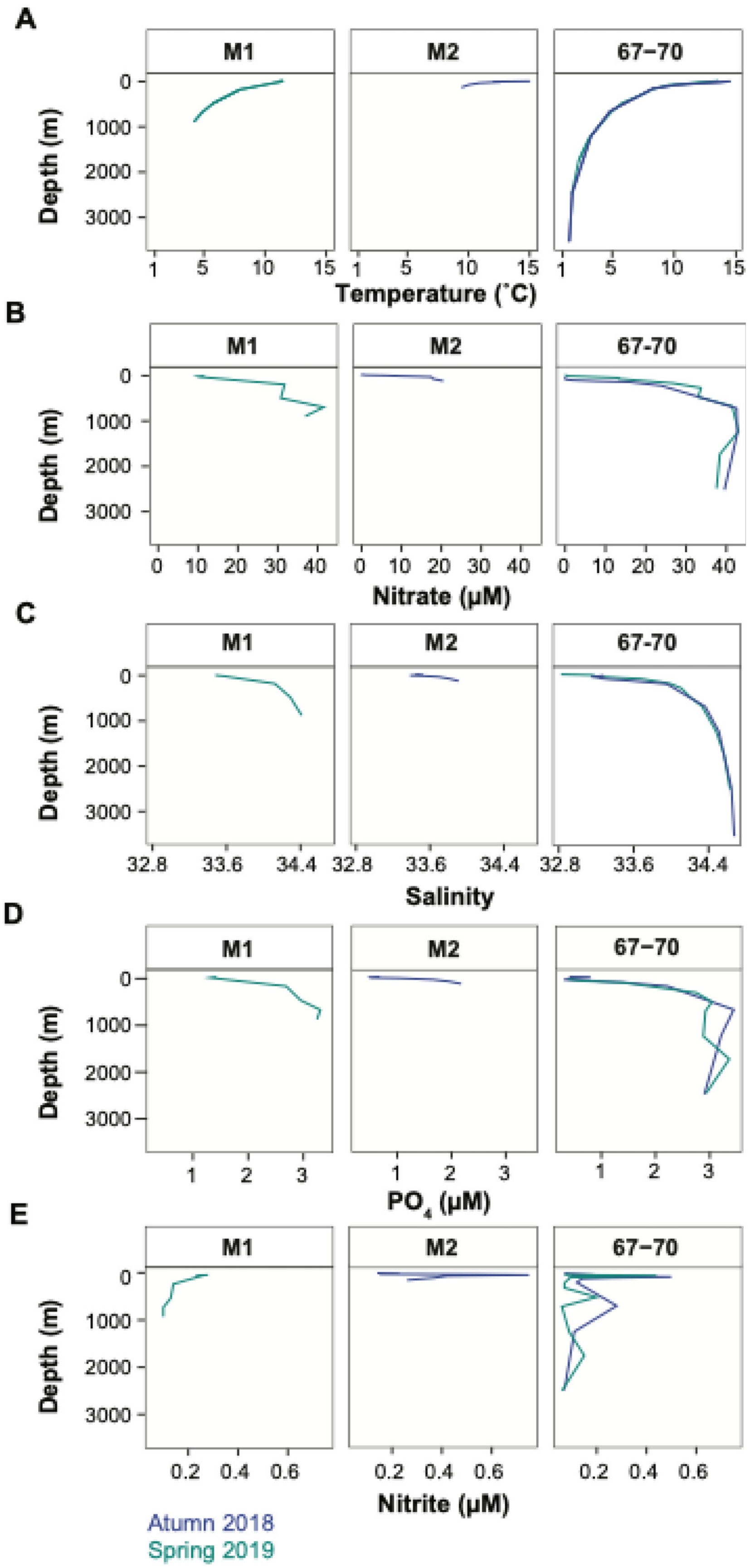
A
B
C
D
E
M1
M2
67−70
Depth (m)
Temperature (˚C)
Nitrate (µM)
Salinity
PO4 (µM)
Nitrite (µM)
Atumn 2018
Spring 2019

Fig. S5. Temporal variations in abiotic parameters at stations M1, M2, and 67-70. Vertical and temporal variation in **A)** temperature, **B)** nitrate, **C)** salinity, **D)** phosphate and **E)** nitrite concentrations at the three stations.

Table S1. Marker genes indicative of vitamin B1 biosynthesis, transport, and salvage, including KEGG identifier and EC number.

KO	Symbol	Gene name	EC
K00788	**thiE**	thiamine-phosphate pyrophosphorylase	2.5.1.3
	**THI20**	hydroxymethylpyrimidine/phosphomethylpyrimidine kinase / thiaminase	2.7.1.49
K00878	**thiM**	hydroxyethylthiazole kinase	2.7.1.50
K00939	**adk, AK**	adenylate kinase	2.7.4.3
K00941	**thiD**	hydroxymethylpyrimidine/phosphomethylpyrimidine kinase	2.7.1.49
K00946	**thiL**	thiamine-monophosphate kinase	2.7.4.16
K00949	**thiN, TPK1, THI80**	thiamine pyrophosphokinase	2.7.6.2
K01077	**phoA, phoB**	alkaline phosphatase	3.1.3.1
K01078	**PHO**	acid phosphatase	3.1.3.2
K01662	**dxs**	1-deoxy-D-xylulose-5-phosphate synthase	2.2.1.7
K02062	**thiQ**	Thiamine import ATP-binding protein ThiQ	NA
K02064	**thiB**	Thiamine-binding periplasmic protein	NA
K03146	**THI4, THI1**	cysteine-dependent adenosine diphosphate thiazole synthase	2.4.2.60
K03147	**thiC**	phosphomethylpyrimidine synthase	4.1.99.17
K03148	**thiF**	sulfur carrier protein ThiS adenylyltransferase	2.7.7.73
K03149	**thiG**	thiazole synthase	2.8.1.10
K03150	**thiH**	2-iminoacetate synthase	4.1.99.19
K03151	**thiI**	tRNA uracil 4-sulfurtransferase	2.8.1.4
K03153	**thiO**	glycine oxidase	1.4.3.19
K03707	**tenA**	thiaminase (transcriptional activator TenA)	3.5.99.2
K04487	**iscS, NFS1**	cysteine desulfurase	2.8.1.7
K05307	**THTPA**	thiamine-triphosphatase	3.6.1.28
K06928	**NTPCR**	nucleoside-triphosphatase	3.6.1.15
K06949	**rsgA, engC**	ribosome biogenesis GTPase / thiamine phosphate phosphatase	3.1.3.100
K07251	**thiK**	thiamine kinase	2.7.1.89
K10810	**tenI**	thiazole tautomerase (transcriptional regulator TenI)	5.3.99.10
K10811		thiamine pyridinylase	2.5.1.2
K14153	**thiDE**	hydroxymethylpyrimidine kinase / phosphomethylpyrimidine kinase / thiamine-phosphate diphosphorylase	2.5.1.3
K14154	**THI6**	thiamine-phosphate diphosphorylase / hydroxyethylthiazole kinase	2.5.1.3
K14154	**THI6**	thiamine-phosphate diphosphorylase / hydroxyethylthiazole kinase	2.7.1.50
K16785	**ykoC**	thiamine permease protein YkoC	NA
K16786, K16787	**ykoD**	thiamine permease protein YkoD	NA

K16789	**thiT**	Thiamine transporter ThiT	NA
K16925	**ykoE**	thiamine permease protein YkoE	NA
K18278	**THI5**	pyrimidine precursor biosynthesis enzyme	NA
K21219	**thiDN**	hydroxymethylpyrimidine kinase / phosphomethylpyrimidine kinase / thiamine-phosphate diphosphorylase	2.5.1.3
K21220	**thiN**	thiamine-phosphate diphosphorylase	2.5.1.3
K22699	**thi4**	sulfide-dependent adenosine diphosphate thiazole synthase	2.4.2.59

Abbreviation: KO; K number representing a functional ortholog that corresponds to a KEGG pathway node or a BRITE hierarchy node.

SI References

1. Wickham H. Ggplot2: elegant graphics for data analysis. Dordrecht ;New York: Springer; 2009.
2. Camacho C, Coulouris G, Avagyan V. *et al*. BLAST+: architecture and applications. BMC Bioinformatics. 2009;10:1-9.
3. Chaumeil P, Mussig A. GTDB-Tk: a toolkit to classify genomes with the Genome Taxonomy Database. Bioinformatics. 2020;36(6):1925-7.
4. Chavez FP. Forcing and biological impact of onset of the 1992 El Niño in central California. Geophys Res Lett. 1996;23(3):265-8.
5. Xiu P, Chai F, Curchitser EN. *et al*. Future changes in coastal upwelling ecosystems with global warming: The case of the California Current System. Sci Rep. 2018;8(1):2866.
6. Reji L, Tolar BB, Chavez FP. *et al*. Depth-differentiation and seasonality of planktonic microbial assemblages in the Monterey Bay upwelling system. Front Microbiol. 2020;11:1075.
7. Qin W, Heal KR, Ramdasi R. *et al*. *Nitrosopumilus maritimus* gen. nov., sp. nov., *Nitrosopumilus cobalaminigenes* sp. nov., *Nitrosopumilus oxyclinae* sp. nov., and *Nitrosopumilus ureiphilus* sp. nov., four marine ammonia-oxidizing archaea of the phylum Thaumarchaeota. Int J Syst Evol Microbiol. 2017;67(12):5067-79.
8. Callahan BJ, McMurdie PJ, Rosen MJ. *et al.* DADA2: High-resolution sample inference from Illumina amplicon data. Nature methods. 2016;13(7):581-3.
9. Saw JHW, Nunoura T, Hirai M. *et al*. Pangenomics analysis reveals diversification of enzyme families and niche specialization in globally abundant SAR202 Bacteria. MBio. 2020;11(1).

Legends for Datasets provided as Excel files:

Supplementary Data S1. Contextual information of CTD profiles (conductivity, temperature, depth, oxygen, and salinity) collected at 42 hydrographic stations (Autumn 2018, and Spring 2019), as well as inorganic nutrient and TRC concentrations. Blank cells mean that the parameter was not measured.

Supplementary Data S2: Summary of seven metagenomic data sets collected during FSEL18 (autumn) and MMV19 (spring) cruises.

Supplementary Data S3: Metagenome Assembled Genomes (MAGs) assembled from ENP metagenomes before dereplication. Bin taxonomy was estimated using gtdb-Tk (v.1.0.2) with default settings and gtdb version 2.14.1 as the reference database. Completeness and contamination estimates were computed using CheckM (v.1.1.3).

Supplementary Data S4: Average rpkm of B1 *de novo* biosynthesis genes in metagenomic (metaG) and metatranscriptomic (metaT) data sets. Abbreviations: *thiC* (hydroxymethyl pyrimidine synthase); *thiD* (hydroxymethyl pyrimidine (phosphate) kinase); *thiDN* (*thiD* fused with *thiN*; thiamine monophosphate synthase); *thiE* (thiamine phosphate synthase); *thiG/thi4*-type (thiazole synthase).

Supplementary Data S5: Statistics for mapping of our metatranscriptomic assemblies of metatranscriptomic reads (6) mapped against AOA reference genomes (NC_010085.1; NZ_CP007026.1; NZ_CP026993.1; NZ_CP026994.1; NZ_CP026995.1) reported to produce B1 *de novo* (7). Expression values are provided in rpkm.

Supplementary Data S6: Metatranscriptome assemblies from (6) mapped against ENP MAGs. Expression values are provided as percent of total rpkm. **S6A:** Number and percent of reads recruited against bacterial and archaeal phyla assigned using gtdb-tk. **S6B:** MAGs used for recruitment analyses; taxonomic affiliation based on gtdb-tk (*see* Material and Methods); and metatranscriptome mapping of data from M1 and M2, percent (%) of total rpkm.

Supplementary Data S7: Summary of amplicon data obtained at 42 hydrographic stations occupied during autumn 2018 (At) and spring cruises 2019 (Sp).

Supplementary Data S8: Summary of bacterial and archaeal Amplicon Sequence Variants (ASVs) representing >1% of reads per sample with their corresponding taxonomic assignment using the dada2 package (v1.18.0; (8)) and Silva_138 reference database.

Supplementary Data S9: Functional annotations of B1-related genes, indicative of *de novo* synthesis, salvage and transport pathways of interest. Annotations include (if available) cog, kegg, eggNOG, pfam, tigrfam, IPR domains and blast hits against ncbi's NR. Data are published on figshare [10.6084/m9.figshare.29133767]

Supplementary Data S10: List of ENP MAGs, encoding marker enzymes for energy generation and carbon fixation in marine Archaea and Bacteria. Functional annotations were obtained using prokka (v1.12). In addition, the blastKOALA and ghostKOALA tool servers were used to obtain KO annotations for predicted proteins (*see* Material and Methods). MAGs encoding also thiamine-

related enzymes (published on figshare [10.6084/m9.figshare.29133767]) have colored background that corresponds to phylum coloring used in the main manuscript.

Supplementary Data S11: Reference genome assemblies of *Nitrospina* and *Thioglobus*-like bacteria, and type strains of ammonia-oxidizing Archaea included in the analysis of genome synteny. Reference genome ID's, amino acid identity (AAI) values and corresponding protein identifiers (sequence IDs; published on figshare [10.6084/m9.figshare.29133767]) used for the analyses presented in Supplementary Figure 2.

Supplementary Data S12: Analysis of thiamine biosynthesis indicator genes in relevant published SAR202 Single Amplified Genome (SAG) and MAG (9). Data includes genome size, completeness, contamination, G+C content, accession numbers of genomes, environmental metadata, and presence and absence of key thiamine-related enzymes (*thiCGE*).

Supplementary Data S13: Summary of the Indicator Species Analysis (Indval) using R software indicspecies (version 1.7.12).

Supplementary Data S14: Summary of ten previously published metatranscriptomic data sets of ammonia-oxidizing marine archaeal communities from Monterey Bay, California, United States. Information includes hydrographic station, IMG/M taxon OID and NCBI accessions.
